# Tumor Suppressor p53 and MicroRNAs Interaction in Breast Cancer

**DOI:** 10.32604/or.2025.072133

**Published:** 2026-02-24

**Authors:** Marcia Eduarda Viana Luna, Gustavo Jacob Lourenço, Juliana Carron

**Affiliations:** 1 Herminio Ometto Foundation, Araras, São Paulo, 13607-339, Brazil; 2 Laboratory of Cancer Genetics, School of Medical Sciences, University of Campinas, Campinas, São Paulo, 13083-888, Brazil

**Keywords:** Breast neoplasms, tumor suppressor protein p53, microRNAs

## Abstract

This literature review explores the complex interaction between p53 and microRNAs (miRNAs) in the occurrence and progression of breast cancer (BC), the most common and lethal tumor type among women. BC is a multifactorial disease resulting from a combination of genetic and epigenetic alterations in cell DNA, influencing proliferation, differentiation, and migration. *TP53* gene, which codifies p53 protein, is a known tumor suppressor, and it plays an important role in cell maintenance as DNA repair, cell proliferation control, and apoptosis activation. *TP53* expression can be modulated by several miRNAs, as miR-30c, miR-34a, and the miR-200 family, inhibiting p53 production and silencing its tumor suppressor effects. On the other hand, p53 protein can modulate several miRNAs expression, as miR-146a, miR-192, and the miR-200 family, by acting as a transcription factor or by modulating miRNA processing, interfering with BC aggressiveness and progression. Understanding the role of p53 and miRNAs in BC may aid in identifying new biomarkers and developing new targeted therapies for patient treatment.

## 1 Introduction

Worldwide, breast cancer (BC) is the most common and most lethal tumor type among women, accounting for 23.8% of all cancer cases and 15.4% of deaths due to the disease [1]. The male population can also develop BC; however, it is less common, accounting for less than 1% of all BC cases [2].

BC can be classified according to the TNM system from the American Joint Committee on Cancer (AJCC) [3] and by its histopathological aspects, especially as invasive ductal, lobular, and tubular carcinoma [4]. In addition, BC classification is defined by the expression of hormone receptors and growth factors, as estrogen receptor (ER), progesterone receptor (PR), and epidermal growth factor receptor 2 (HER-2/ERBB2) [5]. The luminal A subtype (ER and PR positive, HER2 negative) is the most common molecular form, accounting for up to 50% of cases, and is generally associated with a less aggressive profile and better disease prognosis [6]. Luminal B tumors (ER, PR, and HER2 positive or negative) tend to be more aggressive than luminal A due to higher cellular proliferation rates and represent approximately 20% of cases [6]. HER2 positive tumors account for about 15% of cases and are associated with higher recurrence rates; however, they are highly responsive to anti-HER2 targeted therapy [6]. On the other hand, triple-negative or basal-like tumors are considered to have the worst prognosis, as they lack specific therapeutic targets and do not respond to current treatment options [6]. Triple-negative tumors comprise around 15% of cases and are commonly associated with genetic mutations [6].

BC-related cells comprise not only malignant epithelial cells but also a heterogeneous population of non-tumoral cells that constitute the tumor microenvironment (TME). The TME plays a critical role in BC development and progression, being composed of immune, stromal, and endothelial cells, and the extracellular matrix (ECM) [7]. Immune infiltrates include both lymphoid and myeloid-derived populations that mediate innate and adaptive immune responses. Cancer-associated fibroblasts (CAFs) establish reciprocal communication with tumor cells, promoting morphological and mechanical changes that enhance tumor invasiveness. Tumor-associated endothelial cells (TECs) contribute to angiogenesis and interact with other TME components to support vascular stability. Additionally, dynamic remodeling of the ECM by proteolytic enzymes facilitates tumor cell migration, invasion, and metastasis, underscoring the importance of TME in shaping BC behavior [7].

Patients’ therapeutic depends on the clinical, histopathological, and molecular characteristics of the tumor and may include surgery, chemotherapy, radiotherapy, endocrine and/or immunotherapy [8]. The survival rate ranges from 80% for early tumor diagnosis to 10% for late-stage detection [9,10].

The development of BC is multifactorial. Risk factors for BC include increasing age, reproductive characteristics, contraceptive and hormone replacement therapy, BC family history, alcoholism, smoking, and physical inactivity [11]. In addition to the environmental risk factors, genetic and epigenetic predisposition may also influence BC risk and progression [11]. Single-nucleotide variant (SNV), copy number variation (CNV); histone modification, DNA methylation, and post-transcriptional regulation by microRNAs (miRNAs) are important genetic and epigenetic, respectively, alterations in BC [11–13].

Breast cancer 1 (*BRCA1*) and breast cancer 2 (*BRCA2*) are well-known mutated genes in BC [11,14]. In addition, other genes are also associated with BC, as ataxia telangiectasia mutated (*ATM*), cadherin 1 (*CDH1*), *CHEK2* (checkpoint kinase 2), phosphatase and tensin homolog (*PTEN*), and tumor protein p53 (*TP53*) [11,14]. Between them, *TP53* gene is frequently altered in BC and may interfere directly with patients’ treatment success [14].

Current studies have presented substantial evidence that *TP53* participates in a complex gene regulatory network involving miRNAs, which plays critical roles in BC development, progression, and response to therapy [15]. *TP53* gene expression can be modulated through the binding of specific miRNAs, leading to changes in the levels and activity of the p53 protein, encoded by *TP53*. Conversely, the p53 protein can transcriptionally regulate a variety of miRNAs, thereby influencing multiple cellular processes, including cell proliferation, DNA repair, apoptosis, and metastasis [15]. This bidirectional regulation highlights the feedback loops that exist between *TP53* and miRNAs, demonstrating their importance in maintaining cellular homeostasis and in tumorigenesis. In this review, we focused on this complex regulatory network of *TP53* and miRNAs in BC, aiming to summarize current knowledge on their interactions and to discuss their potential implications for prognostic and therapeutic strategies.

## 2 *TP53* Gene

*TP53* codifies the tumor suppressor protein p53 that is activated in response to various cellular stresses, as lack of nutrients, hypoxia, and DNA damage [16]. The tumor-suppressor activity of p53 is mainly attributed to its ability to modulate the expression of several genes involved in cell cycle, DNA repair, apoptosis, senescence, and cell metabolism, in order to protect the DNA and maintain the tissue homeostasis [16].

The p53 belongs to a multiprotein family of transcription factors that also includes p63 and p73. p53 is a phosphorylation target of the kinases ATM, ataxia telangiectasia and Rad3-related (ATR), checkpoint kinase 1 (CHEK1), and CHEK2, which act in coordination to promote its stabilization [17]. These post-translational modifications play a crucial role in stabilizing p53 by disrupting its interaction with the negative regulators double minute 2 protein (MDM2) and double minute 4 protein (MDM4) [17]. MDM2 and MDM4 bind to the transactivation domains of p53, thereby suppressing its transcriptional activity. In addition, MDM2 functions as an E3 ubiquitin ligase that targets p53 for proteasome-dependent degradation [17]. In normal cells, p53 is kept at low levels by MDM2, which is transcriptionally regulated by p53, forming a negative-feedback loop [18]. Additionally, p53 can function as a transcriptional repressor, notably downregulating genes such as transcription factor AP-1 subunit C-Fos (FOS), MYC proto-oncogene, bHLH transcription factor (*MYC*), vascular endothelial growth factor A (*VEGFA*), and *BIRC5* (survivin), inhibiting proliferation, survival, and angiogenesis [18].

*TP53* importance in tumor progression is demonstrated by the high frequency of mutations found in several types of cancer, including BC [19]. SNVs are the most common mutation in *TP53* gene, and it can influence protein-DNA binding domains (as p53^R248Q^ and p53^R273H^ mutations) or cause a partial or total distortion of the correct folding of p53 protein (as p53^R175H^ and p53^H179R^ mutations) [19].

SNVs in *TP53* tend to accumulate in tumor cells, leading to the loss of tumor suppressor functions [19]. Besides, SNVs in *TP53* may also favor BC progression, increasing proliferation, inflammation, angiogenesis, invasion, and chemotherapy resistance, and inhibiting apoptosis, through the called gain-of-function [20].

In addition, *TP53* expression can be mediated by miRNAs binding [15]. The interaction of miRNAs and *TP53* messenger RNA (mRNA) may decrease or even silence the production of p53 protein [15]. On the other hand, p53 protein can also regulate the expression of several miRNAs [14,20]. p53 can interact directly to the promoter region of miRNAs genes or by binding to Drosha complex, responsible for miRNAs maturation, influencing their transcription [15,21]. This complex interaction of *TP53* and miRNAs impacts the occurrence and progression of BC [22–24].

## 3 miRNAs

miRNAs are endogenous small non-coding RNAs with approximately 22 nucleotides that silence gene expression post-transcriptionally through degradation or inhibition of genes’ mRNA [25], including those related to BC carcinogenesis [26,27].

In miRNA gene transcription, RNA polymerase II or III will process the primary miRNA (pri-miRNA) in a structure called hairpin [28]. In the nucleus, the pri-miRNA is processed by Drosha complex, forming the precursor miRNA (pre-miRNA), a large double-stranded RNA, which will be transported to the cytoplasm by Exportin-5 [28]. In the cytoplasm, pre-miRNA is cleaved by the ribonuclease Dicer, producing the mature miRNA [28]. The RNA-induced silencing complex (RISC) is responsible for separating the double strand from the mature miRNA, in which one strand is frequently cleaved and discarded and the other, called guide strand, is guided to the target mRNA [28].

Thus, miRNAs can either degrade target mRNA or repress protein translation through complete or partial base complementarity, respectively. In humans, complementarity between miRNAs and their target mRNAs is typically partial, leading to translational repression without degradation of the mRNA strand [29]. miRNAs contain complementary regions that bind primarily to the 3^′^ untranslated region (3^′^-UTR) of target genes, but can also interact with sequences within the 5^′^-UTR and coding regions, exerting silencing effects on gene expression [28]. Moreover, miRNAs’ interaction with promoter regions has been reported to induce transcription, highlighting their capacity to regulate gene expression through diverse and context-dependent mechanisms [30].

In the context of cancer, miRNAs could be divided into two groups: oncogenic miRNAs or oncomiRNAs, and tumor suppressive miRNAs, depending on their target genes and the resulting effects on tumor development. OncomiRNAs promote tumor progression by negatively regulating tumor suppressor genes that control cell survival, differentiation, apoptosis, invasion, and migration. In contrast, tumor suppressive miRNAs inhibit tumor progression by negatively regulating oncogenes that promote cell survival, differentiation, apoptosis, invasion, and migration [31].

## 4 *TP53* Expression Modulated by miRNAs

Some miRNAs can bind directly to the *TP53* mRNA, inhibiting p53 production and favoring BC development and progression, acting as oncomiRNAs [31].

Reports from the literature have demonstrated that miR-214 [32], miR-504 [33], miR-663a [34], and miR-1204 [35] can bind to the *TP53* mRNA, inhibiting gene expression and increasing cell proliferation and invasion, and decreasing apoptosis of BC cells. In addition, miR-105, miR-200c, miR-659, miR-662, and miR-921 were associated with decreased *TP53* expression, which may contribute to increased invasion and migration of BC cells [36].

**Figure 1:**
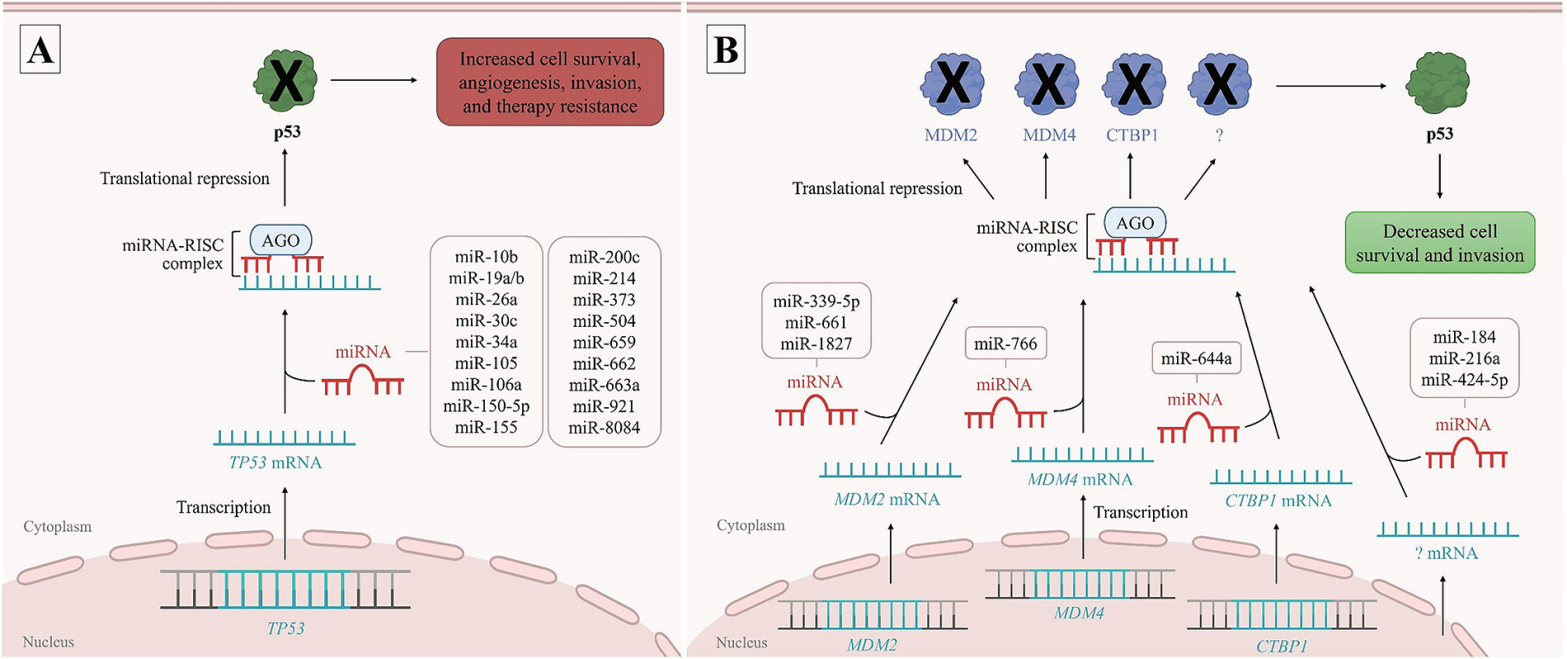
*TP53* expression modulated by microRNAs (miRNAs). Several miRNAs can bind directly to the *TP53* messenger RNA (mRNA), inhibiting p53 production and favoring breast cancer (BC) development and progression, acting as oncomiRNAs (**A**). Other miRNAs can regulate *TP53* expression indirectly by binding and suppressing *TP53* negative regulators, as *MDM2*, *MDM4*, and *CTBP1* genes, thereby allowing p53 production and acting as tumor suppressor miRNAs (**B**). RISC complex: RNA-induced silencing complex, AGO: argonaute proteins. Figure made in PowerPoint (Microsoft 365) using icons sourced from the public domain

miR-19a, miR-19b [37], miR-106a [38], and miR-8084 [39] were also associated with decreased *TP53* expression, leading to increased cell proliferation and migration, along with decreased apoptosis and sensitivity to cisplatin chemotherapy in BC cell lines. Other oncomiRNAs, as miR-10b, miR-30c, miR-34a, miR-373 [40], miR-26a [41], and miR-155 [42], have been associated with decreased *TP53* expression in BC; however, the exact mechanisms involved in BC carcinogenesis remain unclear. An overview of miRNAs involved in modulating *TP53* is schematically illustrated in Fig. 1A.

In contrast, other miRNAs can regulate *TP53* expression indirectly. These miRNAs can bind to *TP53* negative regulators, thereby allowing p53 production and acting as tumor suppressor miRNAs [31]. Among them, miRNAs miR-339-5p [43], miR-661 [44], miR-1827 [45], miR-766 [46], and miR-644a [47] were associated with *TP53* upregulation by targeting the *TP53* regulators MDM2, MDM4, and CTBP1 (C-terminal binding protein 1), respectively. Other miRNAs, such as miR-184 [48], miR-216a [49], and miR-424-5p [50] may exert similar effects, although the exact *TP53* regulator involved remains unclear. Collectively, these tumor-suppressive miRNAs enhance p53-mediated apoptosis, cell cycle arrest, and inhibition of invasion in BC cells. These associations are schematically illustrated in Fig. 1B.

Interestingly, miR-193a-5p [51] and miR-3646 [52] were associated with *TP53* downregulation and decreased cell proliferation and migration, and increased apoptosis and paclitaxel chemotherapy sensitivity. Similarly, miR-150-5p was associated with *TP53* downregulation, but also with decreased tumor progression and improved patient survival [53]. These results counteract the expected effects of low *TP53* expression in BC, and further studies are needed to explain this association. One possible explanation is that these miRNAs may downregulate *TP53* while simultaneously targeting other cancer-related genes, thereby compensating for *TP53* loss.

A comprehensive overview of miRNAs involved in modulating *TP53* expression in BC can be found in Table 1.

**Table 1:** MicroRNAs involved in *TP53* gene regulation in breast cancer (BC)

microRNA	Gene region	Sample type	BC phenotype	*TP53* expression	Final effect	Reference
miR-10b	NR	*In silico* analysis	Luminal A and triple-negative	↓ *TP53*	Uncertain	Rahimi et al. [40]
miR-19a	NR	Cell line	Luminal A and triple-negative	↓ *TP53*	Increased cell proliferation	Li et al. [37]
miR-19b	NR	Cell line	Luminal A and triple-negative	↓ *TP53*	Increased cell proliferation	Li et al. [37]
miR-26a	NR	Cell line	Luminal A and triple-negative	↓ *TP53*	Uncertain	Cabello et al. [41]
miR-30c	NR	*In silico* analysis	Luminal A and triple-negative	↓ *TP53*	Uncertain	Rahimi et al. [40]
miR-34a	NR	*In silico* analysis	Luminal A and triple-negative	↓ *TP53*	Uncertain	Rahimi et al. [40]
miR-105	NR	Cell line	NR	↓ *TP53*	Increased cell invasion and migration	Verma et al. [36]
miR-106a	NR	Tumor tissue	NR	↓ *TP53*	Increased cell proliferation and invasion, decreased apoptosis, and sensitivity to cisplatin	You et al. [38]
miR-150-5p	NR	Tumor tissue	Triple-negative	↓ *TP53*	Decreased tumor aggressiveness	Almohaywi et al. [53]
miR-155	NR	Peripheral blood	Luminal A, B, HER2+, and triple-negative	↓ *TP53*	Uncertain	Liu et al. [42]
miR-184	NR	Cell line	Luminal A	↓ ? = ↑ *TP53*	Probable decrease in cell proliferation and invasion	Feng & Dong [48]
miR-193a-5p	NR	Cell line	Luminal A	↓ *TP53*	Decreased cell proliferation and migration, increased apoptosis, and paclitaxel sensitivity	Khordadmehr et al. [51]
miR-200c	NR	*In silico* analysis	Luminal A and triple-negative	↓ *TP53*	Uncertain	Rahimi et al. [40]
miR-200c	NR	Cell line	NR	↓ *TP53*	Increased cell invasion and migration	Verma et al. [36]
miR-214	3^′^-UTR	Cell line	NR	↓ *TP53*	Increased cell invasion	Wang et al. [32]
miR-216a	NR	Cell line	NR	↓ ? = ↑ *TP53*	Decreased cell proliferation and invasion, increased apoptosis	Xie et al. [49]
miR-339-5p	NR	Cell line	Luminal A	↓ *MDM2* = ↑ *TP53*	Decreased cell proliferation	Jansson et al. [43]
miR-373	NR	*In silico* analysis	Luminal A and triple-negative	↓ *TP53*	Uncertain	Rahimi et al. [40]
miR-424-5p	NR	Cell line	Triple-negative	↓ ? = ↑ *TP53*	Increased apoptosis and sensitivity to paclitaxel	Dastmalchi et al. [50]
miR-504	3^′^-UTR	Cell line	Luminal A	↓ *TP53*	Increased cell proliferation and decreased apoptosis	Hu et al. [33]
miR-644a	NR	Cell line and tumor tissue	Luminal A, B, HER2+, and triple-negative	↓ *CTBP1* = ↑ *TP53*	Decreased cell proliferation and increased apoptosis	Raza et al. [47]
miR-659	NR	Cell line	NR	↓ *TP53*	Increased cell invasion and migration	Verma et al. [36]
miR-661	NR	Cell line	Luminal A, HER2+, and triple-negative	↓ *MDM2* = ↑ *TP53*	Decreased proliferation of cells without TP53 mutation	Hoffman et al. [44]
miR-662	NR	Cell line	NR	↓ *TP53*	Increased cell invasion and migration	Verma et al. [36]
miR-663a	3^′^-UTR	Cell line	Luminal A	↓ *TP53*	Increased cell survival and decreased apoptosis	Cho et al. [34]
miR-766	NR	Tumor tissue	NR	↓ *MDM4* = ↑ *TP53*	Decreased cell proliferation	Wang et al. [46]
miR-921	NR	Cell line	NR	↓ *TP53*	Increased cell invasion and migration	Verma et al. [36]
miR-1204	NR	Cell line	Luminal A	↓ *TP53*	Decreased proliferation and increased apoptosis	Neshastehriz et al. [35]
miR-1827	NR	Cell line	Luminal A	↓ *MDM2* = ↑ *TP53*	Increased apoptosis	Zhang et al. [45]
miR-3646	NR	Cell line	Luminal A and triple-negative	↓ *TP53*	Decreased cell proliferation	Tao et al. [52]
miR-8084	NR	Cell line	NR	↓ *TP53*	Decreased apoptosis	Gao et al. [39]

Note: NR, not reported; 3^′^-UTR, 3^′^ untranslated region; HER2+, HER-2 positive; ↑, increased expression; ↓, decreased expression; ?, unknown; =, linked to.

When considering BC subtypes, different miRNAs associated with *TP53* regulation appear to display subtype-specific patterns. For instance, miR-10b [40], miR-19a, miR-19b [37], miR-26a [41], miR-30c, miR-34a, miR-200c, miR-373 [40], and miR-3646 [52] were reported in luminal A and triple-negative BC, whereas miR-184 [48], miR-193a-5p [51], miR-339-5p [43], miR-504 [33], miR-663a [34], and miR-1827 [45] were reported only in luminal A, and miR-150-5p [53] and miR-424-5p [50] only in triple-negative BC. On the other hand, miR-661 [44] was reported in luminal A, HER2-positive, and triple-negative BC, whereas miR-155 [42] and miR-644a [47] were reported in all four subtypes (luminal A, luminal B, HER2-positive, and triple-negative) (Table 1).

These observations indicate that the functional impact of each miRNA on *TP53* regulation may depend not only on its molecular target but also on the intrinsic biology of each BC subtype. Understanding these relationships is particularly critical for triple-negative BC, where the absence of targeted treatment options makes the discovery of novel miRNA–*TP53*–based therapeutic strategies especially valuable.

## 5 miRNAs Expression Modulated by p53

The p53 protein can control the expression level of several miRNAs through two main mechanisms, particularly by acting as a transcription factor and by modulating miRNA processing [15,21]. In addition to regulating protein-coding genes, p53 also modulates the expression of several miRNA genes by directly binding to their DNA sequences and primarily promoting their transcription [54]. Although p53-mediated transcriptional repression has been described in the literature, the mechanisms underlying this process remain less well understood [54].

Beyond transcriptional regulation, p53 also plays a role in the post-transcriptional maturation of miRNAs. It has been demonstrated that p53 directly binds to the DEAD-box RNA helicase p68 (DDX5), facilitating its association with the Drosha complex, which is responsible for miRNAs maturation [54]. Through this interaction, p53 contributes to the conversion of pri-miRNAs into pre-miRNAs, thereby enhancing the production of mature miRNAs [54].

**Figure 2:**
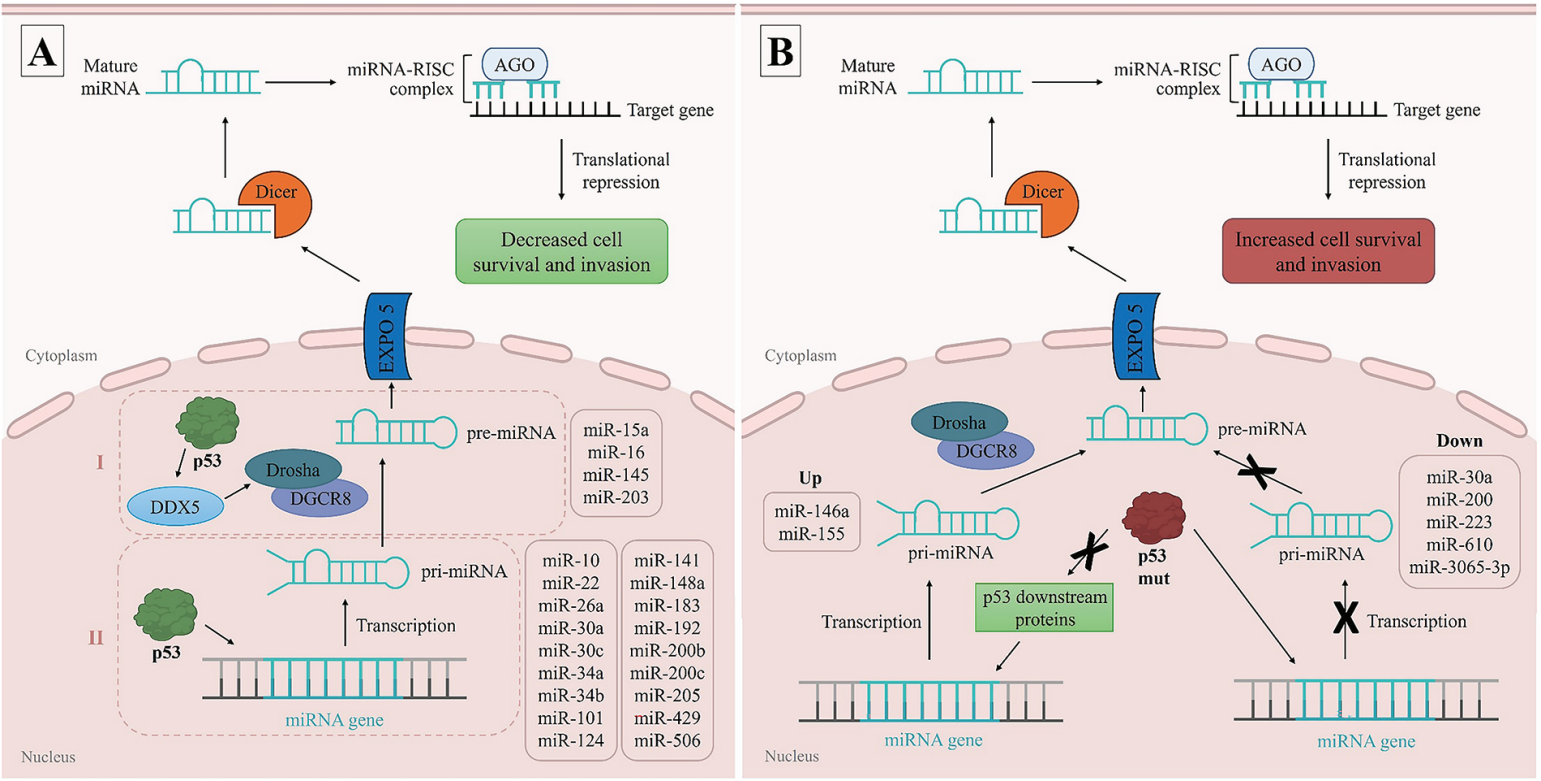
MicroRNAs (miRNAs) expression modulated by p53. The p53 protein can control the expression level of several miRNAs by directly binding to their DNA sequences and primarily promoting their transcription (I), or by interacting with the DEAD-box RNA helicase p68 (DDX5), facilitating its association with the Drosha complex and modulating miRNA processing (II) (**A**). The expression levels of certain miRNAs are influenced by genetic mutations in p53. Mutant p53 (p53 mut), as R273H variant, can bind to promoter regions and repress miRNA expression. On the other hand, p53 mut can also bind to downstream targets of p53, as tumor protein p63, allowing miRNA expression. These dysregulations contribute to the development and progression of BC (**B**). pri-miRNA: primary miRNA, pre-miRNA: precursor miRNA, EXPO5: exportin-5, RISC complex: RNA-induced silencing complex, AGO: argonaute proteins, up: upregulated miRNAs, down: repressed miRNAs. Figure made in PowerPoint (Microsoft 365) using icons sourced from the public domain

In BC, p53 induces the expression of miR-30a [55], miR-34a [56–58], miR-101, miR-124, miR-141 [59], miR-183 [60], miR-192 [59], miR-200b [61], miR-200c [60,62], miR-205 [63], miR-429 [61], and miR-506 [59] by binding to their promoters and inducing transcription. The upregulation of these miRNAs have been associated with the suppression of epithelial–mesenchymal transition (EMT), through the inhibition of transcription factors as Zeb1, Zeb2, and Snail, thereby interfering with the aggressiveness and progression of the disease, acting as tumor suppressive miRNAs [59]. p53 also induces the expression of miR-10, miR-22, miR-26a [22], miR-30c [64], miR-34b [65], and miR-148a [22] by binding to their promoters. These tumor suppressive miRNAs have been associated with a better prognosis in BC by decreasing cell proliferation, invasion, and therapy resistance. In addition, p53 promotes the processing of tumor suppressive miR-15a [23]; miR-16, miR-145, and miR-203 [22] from primary to precursor miRNA, leading to cell apoptosis in response to DNA damage; and decreasing cell proliferation, respectively. An overview of miRNAs expression modulated by p53 is schematically illustrated in Fig. 2A.

Moreover, the expression levels of some miRNAs, such as miR-30a [66], miR-146a [67], miR-155 [68], miR-200 [69], miR-223 [70], miR-610, and miR-3065-3p [71] are influenced by genetic mutations in p53. In BC cells, mutant p53, as the R273H variant, have been observed to bind to the promoter regions and repress the expression of miR-30a [66], miR-200 [69], miR-223 [70], miR-610, and miR-3065-3p [71] thereby increasing cell invasion and migration. On the other hand, the expression of miR-146a [67] and miR-155 [68] was found to be higher in BC cells harboring p53 mutations, likely due to the inactivation of downstream targets of p53, as tumor protein p63 [68]. This dysregulation contributes to increased cell proliferation and chemotherapy resistance, decreased apoptosis, and worse patient prognosis. These associations are schematically illustrated in Fig. 2B.

Finally, miR-10 [22,40], miR-26a [22,41], miR-30c [40,64], miR-34a [40,56], miR-155 [42,68], and miR-200c [40,72] were both regulated by p53 and able to regulate *TP53* gene. This observation evidences the complex regulatory feedback loop between p53 and miRNAs.

A complete approach of miRNAs modulated by p53 in BC can be found in Table 2.

**Table 2:** MicroRNAs regulated by the p53 protein in breast cancer (BC)

MicroRNA	Sample type	BC phenotype	MicroRNA expression	Final effect	Reference
miR-10	*In silico* analysis	NR	↑ miR-10	Decreased cell proliferation	Zhou et al. [22]
miR-15a	Cell line	Luminal A and triple-negative	↑ miR-15a	Increased apoptosis	Yang et al. [23]
miR-16	*In silico* analysis	NR	↑ miR-16	Decreased cell proliferation	Zhou et al. [22]
miR-22	*In silico* analysis	NR	↑ miR-22	Decreased cell proliferation	Zhou et al. [22]
miR-26a	*In silico* analysis	NR	↑ miR-26a	Decreased cell proliferation	Zhou et al. [22]
miR-30a	*In silico* analysis and cell line	Triple-negative	↑ miR-30a	Decreased tumor aggressiveness	Di Gennaro et al. [55]
miR-30a	Tumor tissue	Triple-negative	↓ miR-30a (p53**mut**)	Increased cell migration	Guo et al. [66]
miR-30c	Cell line	Luminal A, B, and triple-negative	↑ miR-30c	Decreased resistance to doxorubicin and better prognosis	Lin et al. [64]
miR-34a	*In silico* analysis and cell line	NR, Luminal A	↑ miR-34a	Decreased cell proliferation and invasion	Zhou et al. [22], Avtanski et al. [56], Hargraves et al. [58]
miR-34a	Tumor tissue	Luminal A, B, HER2+, and triple-negative	↑ miR-34a	Increased cell proliferation and better prognosis	Peurala et al. [57]
miR-34b	Cell line	HER2+	↑ miR-34b	Decreased cell proliferation	Lee et al. [65]
miR-101	Cell line	NR	↑ miR-101	Decreased cell invasion and migration	Parfenyev et al. [59]
miR-124	Cell line	Luminal A and triple-negative	↑ miR-124	Decreased cell proliferation and invasion	Parfenyev et al. [59]
miR-145	*In silico* analysis	NR	↑ miR-145	Decreased cell proliferation	Zhou et al. [22]
miR-146a	Cell line	Luminal A and triple-negative	↑ miR-146a (p53**mut**)	Increased cell proliferation and decreased apoptosis	Sandhu et al. [67]
miR-148a	*In silico* analysis	NR	↑ miR-148a	Decreased cell proliferation	Zhou et al. [22]
miR-155	Cell line	Luminal B and triple-negative	↑ miR-155 (p53**mut**)	Increased cell invasion and migration	Neilsen et al. [68]
miR-183	Cell line	NR	↑ miR-183	Decreased cell invasion and migration	Chang et al. [60]
miR-192	Cell line	Luminal A and triple-negative	↑ miR-192	Decreased cell invasion and migration	Parfenyev et al. [59]
miR-200	Cell line	Luminal A	↓ miR-200 (p53**mut**)	Increased cell invasion and migration	Alam et al. [69]
miR-200b and miR-200c	*In silico* analysis and cell line	NR	↑ miR-200b/c	Decreased cell proliferation	Zhou et al. [22], Tamura et al. [61]
miR-200c	Cell line	Luminal A and triple-negative	↑ miR-200c	Decreased cell invasion and migration	Chang et al. [60], Kumar et al. [62], Chao et al. [72]
miR-203	*In silico* analysis	NR	↑ miR-205	Decreased cell proliferation	Zhou et al. [22]
miR-205	*In silico* analysis and cell line	Triple-negative	↑ miR-205	Decreased cell proliferation, invasion and migration	Zhou et al. [22], Piovan et al. [63]
miR-223	Cell line	HER2+ and triple-negative	↓ miR-223 (p53**mut**)	Increased resistance to chemotherapy	Masciarelli et al. [70]
miR-429	Cell line	NR	↑ miR-429	Decreased cell proliferation	Tamura et al. [61]
miR-506	Cell line	Luminal A and triple-negative	↑ miR-506	Decreased cell proliferation, invasion and migration	Parfenyev et al. [59]
miR-610	*In silico* analysis	Luminal A, B, HER2+, and triple-negative	↓ miR-610 (p53**mut**)	Worse prognosis	Zhang et al. [71]
miR-3065–3p	*In silico* analysis	Luminal A, B, HER2+, and triple-negative	↓ miR-3065–3p (p53**mut**)	Worse prognosis	Zhang et al. [71]

Note: NR, not reported; ↑, increased expression; HER2+, HER-2 positive; ↓, decreased expression; p53mut, mutated p53.

When considering BC subtypes, miR-15a [23], miR-124 [59], miR-146a [67], miR-192 [59], miR-200c [60,62,72], and miR-506 [59] modulation were reported in luminal A and triple-negative BC, whereas miR-200 [69] was reported only in luminal A, and miR-30a [55] and miR-205 [22,63] only in triple-negative BC. The modulation of miR-30c [64] was reported in luminal A, luminal B, and triple-negative BC; and miR-155 [68] in luminal B and triple-negative BC. miR-34b [65] modulation was reported in HER2-positive BC, whereas miR-223 [70] was reported in HER2 positive and triple-negative, and miR-34a [57], miR-610, and miR-3065–3p [71] were reported in all four subtypes (luminal A, luminal B, HER2 positive, and triple-negative) (Table 2).

It is important to highlight that this distribution may not be definitive, as future studies using other BC cell lines or patient cohorts may reveal that these miRNAs are also present in additional BC subtypes, thereby refining our current understanding of their subtype specificity.

## 6 miRNA-Based Therapies Targeting the p53-miRNA Axis

miRNA-based therapy is an emerging approach that aims to regulate gene expression by targeting miRNAs and is being studied in a variety of conditions. By correcting miRNA imbalances, this strategy holds promise for the treatment of several diseases, including BC [73]. Drug resistance and tumor relapse are major factors that compromise the survival of BC patients, underscoring the need for alternative therapeutic approaches to treat BC [73].

miRNA therapeutics are oligonucleotides capable of inhibiting oncogenic miRNAs or replacing tumor suppressive miRNAs. In the last decades, some miRNAs have been assessed in clinical studies, especially as biomarkers for diagnosis and prognosis. However, miRNA-based therapies have not yet led to any approvals by regulatory agencies, and their use in BC treatment remains an emerging field [73]. For example, miR-16 mimics (mesomiR-1 by TargomiRs/EnGeneIC), miR-34 mimics (MRX34 by Mirna Therapeutics), and miR-155 antagomir (MRG-106 by miRagen Therapeutics) have entered clinical trials for oncological diseases, but not for BC [73].

In p53-miRNA axis, miR-26a, miR-34a, miR-203, miR-205; miR-214, and miR-223 were described as potential antitumor or antimetastatic miRNA-based therapeutics, respectively, in animal models [73]. Along with presenting more consistent preclinical data, the development of miRNA-based therapies depends on overcoming major hurdles such as molecule stability and targeted delivery. *In vivo* application of miRNA molecules faces additional challenges, including poor bioavailability, enzymatic degradation, rapid clearance, and inefficient cellular uptake, which also need to be further explored [74].

In addition, although p53 does not possess typical drug target features, several strategies to manipulate p53 in cancer have been developed in recent years, including reactivation of mutant p53, induction of mutant p53 degradation, and exploitation of synthetic lethality in p53-deficient cells. Some of these drugs, such as eprenetapopt (APR-246), COTI-2, arsenic trioxide, nutlin-3a, and PC14586, are currently under evaluation in clinical trials, with the most advanced being eprenetapopt, primarily in myelodysplastic syndrome [75,76]. In BC, these p53-targeting drugs have not yet produced conclusive clinical results. Some strategies have been explored preclinically, particularly in triple-negative BC, such as zoledronic acid and atorvastatin, which may promote degradation of mutant p53 or inhibit pathways enhanced by mutant p53 [75,76]. No p53-based therapy has yet reached a clinically validated stage for BC, and most approaches remain experimental. Once available for BC, these therapies could potentially influence the p53–miRNA axis. Indeed, in a BC study, eprenetapopt was shown to restore the transcriptional activity of mutant p53, which in turn leads to the upregulation of miR-30c [77], a miRNA involved in mediating p53-dependent tumor suppressive effects [40,64].

## 7 Perspectives

The regulatory interplay between p53 and miRNAs represents a highly intricate network with critical implications for BC biology. Despite significant advances in our understanding of this axis, several knowledge gaps and methodological limitations remain that hinder its full translational potential.

One of the key challenges lies in the context-dependent behavior of specific miRNAs, which often produce outcomes that diverge from expected canonical effects. For instance, miR-193a-5p [51], miR-3646 [52], and miR-150-5p [53] have been reported to downregulate *TP53* expression, yet paradoxically exhibit anti-proliferative and pro-apoptotic effects, along with increased chemosensitivity and improved patient survival. These findings challenge the conventional expectation that downregulation of *TP53* necessarily promotes tumor progression. Moreover, miRNAs such as miR-10 [22,40], miR-26a [19,41], miR-30c [40,64], miR-34a [36,56], miR-155 [42,68], and miR-200c [36,72] are not only regulated by p53 but also regulate *TP53* expression themselves, forming feedback loops that may shift functional outcomes depending on p53 status (wild-type vs. mutant) [78], epigenetic modulation [79], or external stress signals [80]. These regulatory paradoxes underscore the need for functional studies that integrate transcriptomic, proteomic, and phenotypic data.

In addition to biological complexity, there are technical limitations in current methodologies. The detection and quantification of circulating miRNAs remain inconsistent across studies due to variability in sample types (e.g., serum vs. plasma), normalization protocols, and analytical platforms [81,82]. This lack of standardization hampers reproducibility and delays the clinical validation of miRNAs as biomarkers or therapeutic agents. Moreover, miRNA-based therapies still face substantial delivery challenges, including low stability in circulation, enzymatic degradation, inefficient cellular uptake, and off-target effects [74].

Emerging technologies offer promising avenues to overcome these barriers. Single-cell RNA sequencing and spatial transcriptomics allow for the dissection of miRNA-p53 interactions at the resolution of individual cells and microenvironments, unveiling heterogeneity that is masked in bulk analyses [83]. CRISPR-based functional screens provide powerful tools to interrogate causal relationships between miRNAs, *TP53*, and downstream pathways in specific BC subtypes [84]. Integrating these approaches with machine learning models may further enable the identification of miRNA signatures predictive of prognosis, therapeutic response, or resistance mechanisms.

## 8 Conclusion

*TP53* and miRNAs play an important and complex role in the gene regulation network in BC, which still requires further study. This knowledge will be extremely important for identifying new biomarkers for the diagnosis and follow-up of the disease and, above all, will assist in the development of potential targeted therapies for patient treatment, especially for those who do not respond to conventional therapy.

## Data Availability

Not applicable.
